# Radiographic and pathologic response of myxoid liposarcoma treated with preoperative radiotherapy

**DOI:** 10.2478/raon-2025-0032

**Published:** 2025-06-16

**Authors:** Robert W Gao, Judith AS Jebastin, Doris E Wenger, William S Harmsen, Andrew L Folpe, Michael G Haddock, Ivy A Petersen, Safia K Ahmed

**Affiliations:** 1 Department of Radiation Oncology, Mayo Clinic, Rochester, Minnesota, USA; 2 Department of Laboratory Medicine and Pathology, Mayo Clinic, Rochester, Minnesota, USA; 3 Department of Radiology, Mayo Clinic, St SW, Rochester, Minnesota, USA; 4 Department of Biostatistics & Information, Mayo Clinic, Rochester, Minnesota, USA; 5 Department of Department of Radiation Oncology, Mayo Clinic, Phoenix, Arizona, USA

**Keywords:** myxoid liposarcoma, sarcoma, radiotherapy, adaptive radiotherapy

## Abstract

**Background:**

We retrospectively assessed volumetric response of myxoid liposarcoma (MLPS) with preoperative radiotherapy (RT) and sought to identify disease and treatment characteristics associated with response.

**Patients and methods:**

We identified all patients with a histologic diagnosis of MLPS who received preoperative RT from 2013 to 2021 at our institution. We used cone beam computed tomography (CBCT) to assess changes in tumor volume and greatest dimension during treatment. Tumors were contoured on CBCT images prior to treatment and at the end of each week of RT. Percentage change in tumor volume and greatest dimension were calculated based on pre-treatment and final week contours. Patients with tumors incompletely visualized on CBCT were excluded from volume analysis but included on greatest dimension analysis. Magnetic resonance imaging (MRI) was used to evaluate pre- and post-RT radiographic features. Surgical pathology was reviewed to record pathologic characteristics.

**Results:**

Twenty patients met inclusion criteria. Most tumors (18/20) were low grade. The most frequent dose/fractionation scheme was 50 Gy in 25 fractions (16/20), with 3 patients receiving 36 Gy in 18 fractions. Median pre-RT volume and greatest dimension were 120 cc (interquartile range [IQR]: 56–399) and 11.2 cm (IQR: 8.4–14.1), respectively. Median percentage change in volume and greatest dimension were -37% (IQR: -57 to -29) and -10% (IQR: -20 to -7). All evaluable tumors decreased in volume during RT. Between pre- and post-RT MRI, most patients had a decrease in intratumoral (16/20) and peritumoral edema (11/20). Sixteen patients exhibited extensive pathologic response. There were no significant associations between radiographic and pathologic features and volumetric change. Local failure at 3 years was 9% (95% confidence interval: 1–59).

**Conclusions:**

We report significant decreases in MLPS tumor size during preoperative RT. There may be a role for adaptive RT planning to reduce target volumes and minimize RT-associated morbidity.

## Introduction

The standard of care for soft tissue sarcoma (STS) of the extremity is surgical resection.^1,2^ In patients undergoing limb-sparing surgery, radiotherapy (RT) is frequently used to decrease the risk of local recurrence.^3–5^ Compared to postoperative RT, preoperative RT involves lower doses and smaller volumes, resulting in fewer late, irreversible complications.^6^ Therefore, preoperative RT is often preferred.^7^ However, acute wound complications are more common with preoperative RT compared to postoperative RT.^8^ Although usually temporary, wound complications have a significant negative impact on quality of life. Considerable efforts have been made to reduce RT-related toxicity by reducing target volumes.^9^

Myxoid liposarcoma (MLPS) is a particularly radiosensitive STS subtype.^10,11^ Whereas a preoperative dose of 50 Gy in 25 fractions is standardly used for extremity STS, early results from the single-arm DOREMY trial demonstrate that deescalation to 36 Gy in 18 fractions for MLPS is safe and effective.^12^ Retrospective series have shown that MLPS tumors treated with preoperative RT undergo significant intratreatment volume reduction.^11,13^ There may be potential to adaptively plan RT during treatment to reduce volumes and minimize treatment-related morbidity. Limited data exist, however, on volume changes during treatment. A previous study evaluating intratreatment volume changes in all STS subtypes included 11 patients with MLPS and showed that only tumors with MLPS histology experienced a decrease in volume.^14^ The rarity of MLPS makes prospective or large-scale study of the disease challenging.

At our large sarcoma referral center, MLPS patients receiving preoperative RT typically undergo onboard cone-beam computer tomography (CBCT) prior to treatment delivery to confirm correct target positioning. CBCT images provide an opportunity to visualize tumor volume throughout treatment. In this retrospective study, we used CBCT to assess intratreatment volumetric changes of MLPS tumors receiving preoperative RT. We also evaluated for radiographic and pathologic factors associated with volumetric change, with the goal of identifying patients most likely to benefit from adaptive RT.

## Patients and methods

### Patients and interventions

Following Institutional Review Board approval (ID 21-004217), we queried a prospectively maintained departmental database to identify all patients with a histologic diagnosis of MLPS of the extremity or trunk. We included those receiving preoperative RT with CBCT imaging available. Neoadjuvant and adjuvant systemic therapy were allowed and were at the discretion of the treating medical oncologist. Acute radiation-related toxicities were recorded within the prospectively maintained database and graded by Common Terminology Criteria for Adverse Events.

A retrospective review of the electronic medical record was performed to collect baseline patient characteristics. Patients received preoperative RT using either 3-dimensional conformal RT or intensity modulated RT based on tumor location relative to organs-at-risk. One patient received intensity modulated proton therapy. Prescription dose/fractionation was recorded and was most often 50 Gy in 25 fractions. For patients receiving a boost, boost dose and fractionation are reported. Intraoperative RT (IORT) or postoperative brachytherapy was allowed and was administered in cases with concern for a positive surgical margin.

Postoperative clinical documentation was reviewed for complications. Acute wound complications were defined as secondary operation under general or regional anesthesia for wound repair or wound management without secondary operation. Late complications were defined as grade 2+ fibrosis, edema, or joint stiffness.^6,8^

### Volumetric assessment

Tumors were contoured using the Varian EclipseTM (Varian Medical Systems, Palo Alto, CA) treatment planning system on CBCT images prior to the first fraction and at the end of each week of RT. Volume and greatest dimension were recorded at each time point. Percentage change in tumor volume and greatest dimension were calculated based on the pre-treatment and final week contours. Patients with tumors incompletely visualized on CBCT were excluded from volume analysis but included on greatest dimension analysis, using the greatest dimension of tumor seen on available CBCT slices.

### Radiographic and pathologic assessment

All patients underwent magnetic resonance imaging (MRI) prior to and following completion of RT. The following features were assessed on MRI: intratumoral enhancement, peritumoral enhancement, peritumoral edema, and fat component. The degree of change in these features was recorded by comparing pre- and post-RT images.

Following RT, patients proceeding to surgical resection. Tumor grading was based on the pre-radiotherapy needle core biopsies, with low-grade MLPS containing by definition less than 5% highly cellular, “round cell” areas. Because formal estimation of the percentage of round cell areas is not always possible on needle biopsies, it is the practice of our specialized sarcoma pathologists to regard as “high-grade” any tumor showing any round cell areas in the biopsy. Our strict criteria for round cell areas includes the presence of cells with high nuclear grade, high cellularity with cellular overlap, identifiable mitotic activity, and obscuring of the delicate capillary pattern seen in low-grade MLPS.

Surgical resection specimens were reviewed for margin status, greatest measurable dimension, percentage of necrosis, and the percentage of viable tumor. “Viable tumor” was used instead of “necrosis,” as treated MLPS typically shows broad zones of fibrosis containing few tumor cells, rather than clearly necrotic sarcoma. Extensive pathologic response was defined as < 50% viable tumor remaining per the DOREMY trial.

### Statistical analysis

Descriptive statistics are reported as number (percentage) for discrete variables and as median (interquartile range, IQR) for continuous variables. The percentage change in volume and greatest dimension were displayed in individual patients across the days of follow-up, along with the mean and 95% confidence interval across all patients. The Kaplan Meier method was used to estimate the 3-year cumulative incidence of local failure, overall survival, and disease-free survival (defined as the earliest occurrence of local failure, distant failure, or all-cause death). All analyses were completed using SAS version 9.4.

## Results

### Patient, radiographic, and pathologic features

Twenty patients were included (Table 1). Median age was 51. The most common tumor site was the proximal lower extremity (13/20). RT was most often delivered using a 3D conformal technique (11/20) to a dose of 50 Gy (16/20). One patient each received IORT (10 Gy) and postoperative high dose rate brachytherapy (28 Gy in 4 fractions).

**TABLE 1.** Patient and radiotherapy characteristics

	N = 20
**Sex**	
Female	8 (40%)
Male	12 (60%)
**Age (years)**	
Median (IQR)	51 (39–60)
**Tumor site**	
Lower extremity, proximal	13 (65%)
Lower extremity, distal	3 (15%)
Trunk	4 (20%)
**Recurrence**	1 (5%)
**Chemotherapy**	
Neoadjuvant	1 (5%)
Adjuvant	1 (5%)
**RT modality**	
3D conformal	11 (55%)
IMRT	8 (40%)
IMPT	1 (5%)
**RT dose**	
36 Gy	3 (15%)
50 Gy	16 (80%)
56.25 Gy	1 (5%)
**IORT**	1 (5%)
**Brachytherapy**	1 (5%)

1 IORT = intraoperative radiotherapy; IQR = interquartile range; IMPT = intensity modulated proton therapy; IMRT = intensity modulated radiotherapy; RT = radiotherapy

Table 2 describes changes in radiographic features based on pre-*versus* post-RT MRI. Most tumors demonstrated a decrease in intratumoral enhancement (16/20) and peritumoral edema (11/20). Of the five tumors with peritumoral enhancement prior to RT, four exhibited a decrease in enhancement.

**TABLE 2.** Radiographic and pathologic characteristics

	N = 20
**Intratumoral enhancement**	
Stable	4 (20%)
Decrease	16 (80%)
**Peritumoral enhancement**	
Increase	1 (5%)
Not present	15 (75%)
Decrease	4 (20%)
**Peritumoral edema**	
Increase	4 (20%)
Stable	5 (25%)
Decrease	11 (55%)
**Fat**	
Increase	11 (55%)
Stable	9 (45%)
**Biopsy grade**	
Low	18 (90%)
High	2 (10%)
**Margin status**	
Negative	18 (90%)
Positive	2 (10%)
**Greatest pathologic dimension (cm)**	
Median (IQR)	9 (7–15)
**Percentage viable tumor**	
Median (IQR)	5 (5–35)
**Extensive pathologic response**	
Median (IQR)	16 (80%)

1 IQR = interquartile range

Pathologic characteristics are listed in Table 2. Eighteen of 20 of tumors were low-grade on nee dle biopsy and 2 were high-grade. Two patients had positive margins, one of whom received IORT. Median percentage of viable tumor was 5% [IQR: 5–35)], with the majority of tumors (16/20) exhibiting extensive pathologic response.

### Volumetric response

**FIGURE 1.**
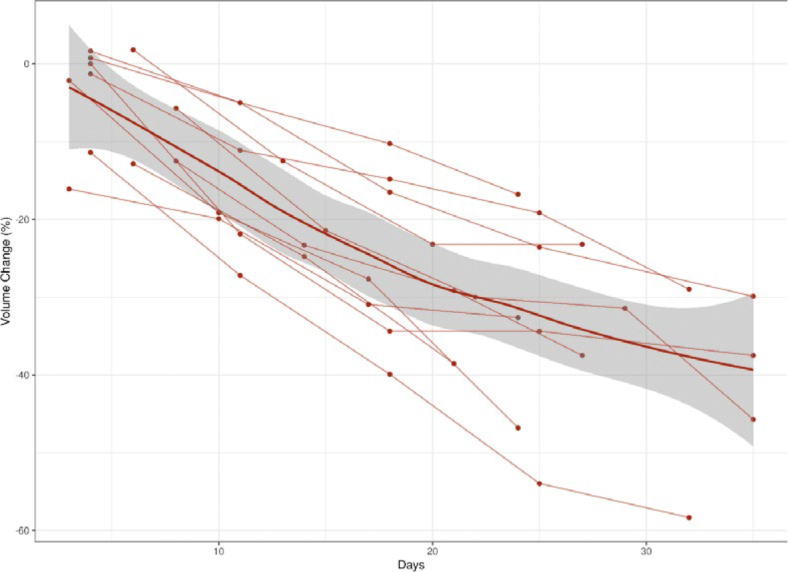
Percentage change in volume during radiotherapy. Dotted lines represent individual tumors. Bold line indicates mean. Gray bar indicates 95% confidence interval.

**FIGURE 2.**
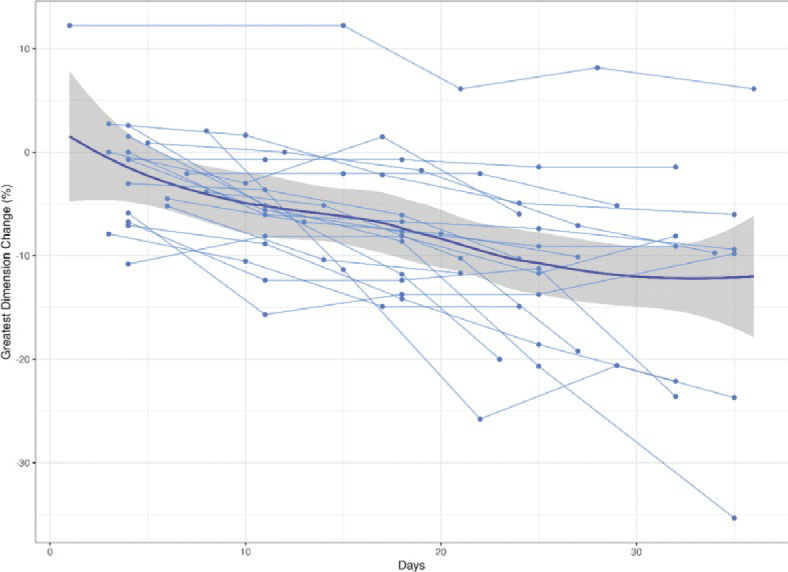
Percentage change in greatest dimension during radiotherapy. Dotted lines represent individual tumors. Bold line indicates mean. Gray bar indicates 95% confidence interval.

Median pre-RT tumor volume and greatest dimension were 120 cc (IQR: 56–399) and 11.2 cm (IQR: 8.4–14.1), respectively. Figures 1 and 2 illustrate percentage change in volume and greatest dimension, respectively, during RT. Eleven tumors were completely visualized on CBCT and assessed for volume change. The median change in tumor volume was -38% (IQR: -57 to -29). All evaluable tumors decreased in volume during treatment. All 20 tumors were assessed for change in greatest dimension. The median change in greatest dimension was -10% (IQR: -20 to -7). Only one tumor demonstrated an increase in greatest dimension.

The radiographic and pathologic characteristics in Table 2 were evaluated for an association with volume change and greatest dimension change. No factors assessed were statistically significant.

### Replan

**FIGURE 3.**
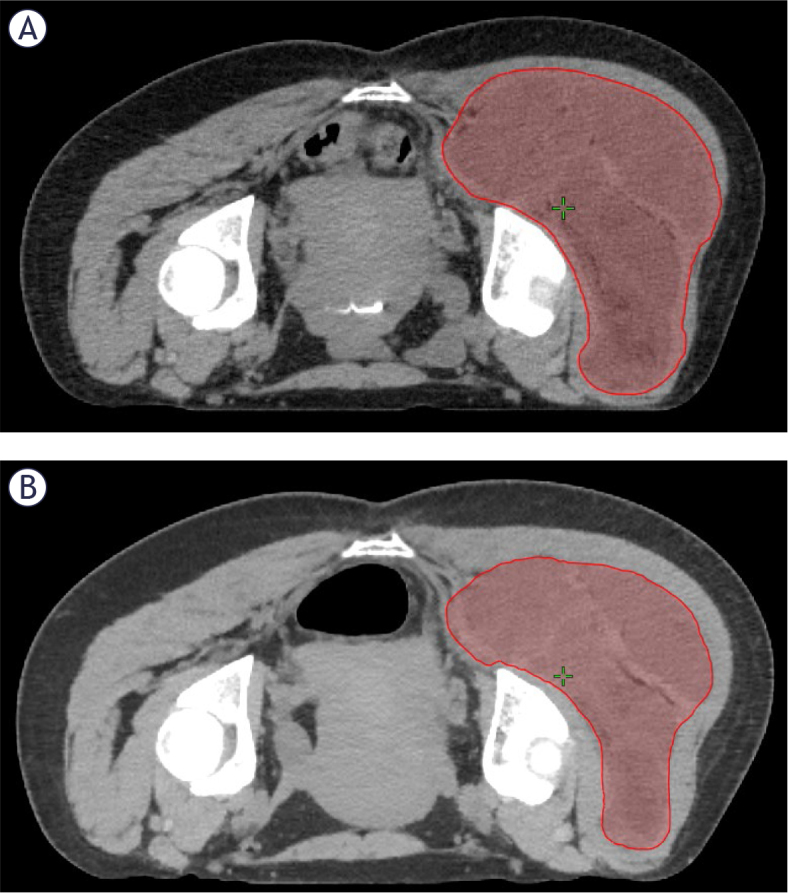
Volumetric change in tumor size from **(A)** prior to treatment to **(B)** following 13 fractions in a patient who underwent mid-radiotherapy replanning. Red contour indicates gross tumor volume. The gross tumor volume decreased from 1780 cc prior to treatment to 1287 cc on the replanning scan.

One patient with an 18.3 cm right gluteal tumor was replanned during treatment due to substantial tumor shrinkage. The prescription dose was 56.25 Gy in 25 fractions. Replanning occurred following 13 of 25 fractions. The contoured gross tumor volume decreased from 1780 cc prior to treatment to 1287 cc on the replanning scan (Figure 3). A dosimetric comparison of the original and new IMRT plans is provided in Table 3. Replanning resulted in improvements in dose to bowel, rectum, genitalia, and the soft tissue avoidance structure. The patient did not experience acute wound complication or late complications following treatment.

**TABLE 3.** Dosimetric comparison of original plan and replan for an individual patient

	Original plan	Replan
**Soft tissue avoid**		
V30Gy(%)	10.3%	0.3%
**Large bowel V45Gy(%)**		
V45Gy(%)	5.4%	1.5%
V50Gy(%)	1.8%	0.4%
**Small bowel**		
V30Gy(cc)	101 cc	82 cc
V45Gy(cc)	13 cc	6 cc
V50Gy(cc)	2 cc	1 cc
**Rectum**		
Mean dose	23 Gy	16 Gy
**Genitalia**		
Mean dose	10 Gy	6 Gy

1 V__Gy = volume receiving at least __Gy

### Oncologic outcomes and complications

At a median follow-up of 2.4 years, the 3-year cumulative incidence of local failure was 9% (95% confidence interval [CI]: 1–59). One patient experienced local failure approximately two years from RT completion. This patient received 50 Gy in 25 fractions to a low grade myxoid liposarcoma of the left thigh. Pretreatment tumor volume was 399 cc and posttreatment volume was 332 cc, for a change of -17%. On MRI, the patient’s tumor exhibited a decrease in intratumoral and peritumoral enhancement and stable peritumoral edema. Pathologic evaluation showed extensive treatment response and 60% necrosis.

Three patients had a distant recurrence during the follow-up period, two in the paraspinal musculature and one in the retroperitoneum. One patient died of non-oncologic causes. Kaplan-Meier estimates of 3-year disease free and overall survival were 60% (95% CI: 38–96) and 92% (95% CI: 76–100), respectively.

Seven patients experienced acute grade 2 radiation-related toxicities, the most common of which were dermatitis (4/20) and edema (3/20). No acute grade 3+ radiation-related toxicities were observed.

Three patients experienced acute wound complications. All three events involved irrigation and debridement of an infected or nonhealing wound. Four patients experienced late complications (two grade 2 fibrosis and two grade 2 edema).

## Discussion

We report our institutional experience of preoperative RT for MLPS. MLPS is a uniquely radiosensitive histology compared to other soft tissue sarcomas. Multiple reports have shown that MLPS exhibits a higher rate and magnitude of tumor reduction with preoperative RT relative to other histologies.^14–17^ Patients treated in our series had a median reduction in tumor volume of 38%. This is consistent with prior studies demonstrating reductions ranging from 19% to 82%.^12–15,17,18^

The unique volumetric kinetics of MLPS make these cases potentially ideal for adaptive replanning. The clinical benefit of replanning varies based on initial tumor size, location, and proximity to organs-at-risk. One patient in our study had replanning performed for a large gluteal tumor. Replanning resulted in notable improvements in dose to bowel, rectum, genitalia, and uninvolved soft tissue, and the patient did not experience any acute or late toxicity. Mid-treatment replanning should be considered for these patients. In addition, CBCT- and MRI-guided online adaptive RT are being increasingly utilized and are appealing options for patients with MLPS.

We used pre- and post-RT MRI to assess radiographic changes. Most tumors showed a decrease in intratumoral enhancement and peritumoral edema following preoperative RT. Similar changes have been observed previously and have been shown to predict for pathologic treatment response^12,19^, however no studies to our knowledge have demonstrated correlation between volumetric change and MRI features. We attempted to identify such features, but none were statistically significant. Houdek *et al*. demonstrated that volume reduction is prognostic; patients in their study with > 25% volume reduction had a 10-year disease specific survival of 86%, compared to 37% among those with ≤ 25% reduction.^20^ Mid-treatment MRI changes have the potential to inform de-escalation or intensification of preoperative RT and adjuvant systemic therapy, and further investigation is needed.

Sixteen of 20 tumors demonstrated major pathologic response. We did not identify an association between volumetric change and pathologic features or pathologic response. Roberge *et al*. investigated the relationship between volumetric and pathologic response and did find a correlation. However, their study included all soft tissue sarcoma histologies.^17^ Prior evidence has also shown that tumor size and grade may predict for patient outcome.^21–25^ A previous analysis at our institution demonstrated that, in patients with MLPS, round cell differentiation and tumor necrosis portend a poor prognosis.^21^ Additional study is warranted to determine if volumetric change during preoperative RT is associated with certain pathologic features.

Our study is limited by its retrospective nature. We did not find radiographic or pathologic variables significantly associated with volumetric change, due to limited patient numbers. Some tumors could not be fully visualized on CBCT, and greatest dimension was used as a surrogate for tumor volume in these cases. MRI is the preferred imaging modality for soft tissue sarcomas^1,2^, however tumor size was assessed on CBCT as intratreatment MRI was not routinely performed.

We describe volumetric change and radiographic and pathologic features in patients with MLPS treated consecutively at our institution with preoperative RT. Tumors demonstrated significant size regression, providing a potential opportunity for adaptive replanning and possible reduction in treatment-associated toxicities. Characteristic radiographic and pathologic changes were observed, although no significant relationship was found with volumetric change.

## Conclusions

We report significant decreases in MLPS tumor size during preoperative RT. There may be a role for adaptive RT planning to reduce target volumes and minimize RT-associated morbidity. We did not identify radiographic or pathologic features associated with volumetric change.
